# The relationship between sedentary behavior, sleep duration, and sleep disorders: analysis of the 2007–2014 National Health and Nutrition Examination Survey

**DOI:** 10.3389/fneur.2025.1488443

**Published:** 2025-06-10

**Authors:** Keke Ju, Na Liu, Ruikai Wu, Xiujiang Shi

**Affiliations:** ^1^Shaanxi Provincial People's Hospital, Xi'an, China; ^2^Department of Gastroenterology, First Affiliated Hospital of Xinjiang Medical University, Urumqi, China

**Keywords:** sedentary behavior, sleep duration, sleep disorders, RCS, subgroup analysis

## Abstract

**Background:**

Sedentary behavior is closely related to sleep disorders, and long-term lack of physical activity may disrupt circadian rhythms and increase the risk of sleep disorders; Excessive or insufficient sleep time may exacerbate health risks, therefore, analyzing the correlation between sedentary behavior, sleep duration, and sleep disorders.

**Methods:**

Using the NHANES research project, multiple logistic regression analysis was conducted to investigate the relationship between sedentary behavior, sleep duration, and sleep disorders in adults. Restrictive cubic spline curves were used to further explore the dose–response relationship between sedentary behavior, sleep duration, and sleep disorders.

**Results:**

In the entire study population, compared to the lowest quartile of Sedentary activity duration, the third quartile [OR = 1.441 (95% CI: 1.226–1.693), *p* < 0.05] and fourth quartile [OR = 1.480 (95% CI: 1.267–1.729), *p* < 0.05] had an increased risk of developing sleep disorders. Compared with adults who sleep for less than 6 h, those who sleep for 6 to less than 8 h [OR = 0.444 (95%CI: 0.395–0.499), *p* < 0.05], ≥8 h [OR = 0.370 (95%CI: 0.325–0.422), *p* < 0.05] the risk of developing sleep disorders decreases. Subgroup analysis found that sedentary behavior and sleep duration have a higher impact on sleep in men under 45 years old. There is a dose–response relationship between sedentary behavior, sleep duration, and the risk of sleep disorders. RCS analysis results show that prolonged sitting for more than 300 min significantly increases the risk of sleep disorders; when the sleep duration is less than 7 h or greater than 8 h, there is a significant increase in the risk of developing sleep disorders. Sensitivity analysis confirmed the robustness of the research results.

**Conclusion:**

There is a significant non-linear relationship between sedentary time, sleep duration, and sleep disorders. It is recommended to limit sedentary time to 300 min per day and sleep duration to 7–8 h to reduce the risk of sleep disorders and improve sleep quality.

## 1 Introduction

Humans spend one-third of their lives sleeping, underscoring the critical role of sleep in overall health. Sleep quality directly impacts quality of life, mental health, and physiological wellbeing (1–7). Research has linked good sleep quality to cardiovascular health, metabolic function, immune system regulation, and cognitive performance (8–10). According to a 2023 World Health Organization (WHO) report, sleep disorders have become the second-largest global mental health issue, affecting approximately 45% of urban populations, with a global prevalence of 27%. Over the past 50 years, average sleep duration among adults has decreased by 1.5 h, and 30–45% of adults report insufficient sleep or poor sleep quality. In the United States alone, sleep disorders result in an annual productivity loss of $54 billion.

The widespread use of electronic devices and the rise of sedentary occupations have made prolonged sitting a common part of modern life. Studies indicate that 60% of jobs require extended periods of sitting, particularly in fields such as information technology, finance, and education. Occupational sedentary time has increased from an average of 3 h per day in 1950 to 8.2 h in 2020, with 30% of adults sitting for at least 6 h daily during weekdays and 37% on weekends (11).

The WHO’s 2020 Guidelines on Physical Activity and Sedentary Behavior define sedentary behavior as any waking activity with an energy expenditure ≤1.5 MET (metabolic equivalent), regardless of posture or duration (12). Common sedentary activities include working, studying, watching television, using computers, reading, writing, driving, and conversing. The WHO ranks sedentary behavior among the top 10 contributors to mortality and disease, associating it with cardiovascular diseases, obesity, diabetes, and other health issues (13–15). Globally, approximately 2 million deaths annually are attributed to sedentary behavior, which is now the fourth-leading risk factor for mortality. Systematic reviews and guidelines have summarized extensive evidence on the adverse health effects of prolonged sitting (16–25). A recent large-scale cohort study published in The Lancet (11) found that sitting for >6 h daily increases the risk of 12 non-communicable diseases, including migraines, rheumatoid arthritis, chronic obstructive pulmonary disease (COPD), chronic liver disease (CLD), diabetes, depression, chronic kidney disease (CKD), asthma, thyroid disorders, gout, diverticulosis, and ischemic heart disease (IHD) (26–31).

Sedentary behavior is closely linked to sleep disorders. Prolonged sitting reduces sleep drive, induces physical discomfort, disrupts metabolism and psychological states, shortens total sleep duration, and alters sleep architecture, thereby contributing to sleep disorders. Improving sedentary habits and increasing physical activity can enhance sleep quality and overall health. This study investigates the relationship between sedentary behavior, sleep duration, and sleep disorders to provide scientific evidence for public health interventions.

## 2 Methods

### 2.1 Data source and participants

**Figure 1:**
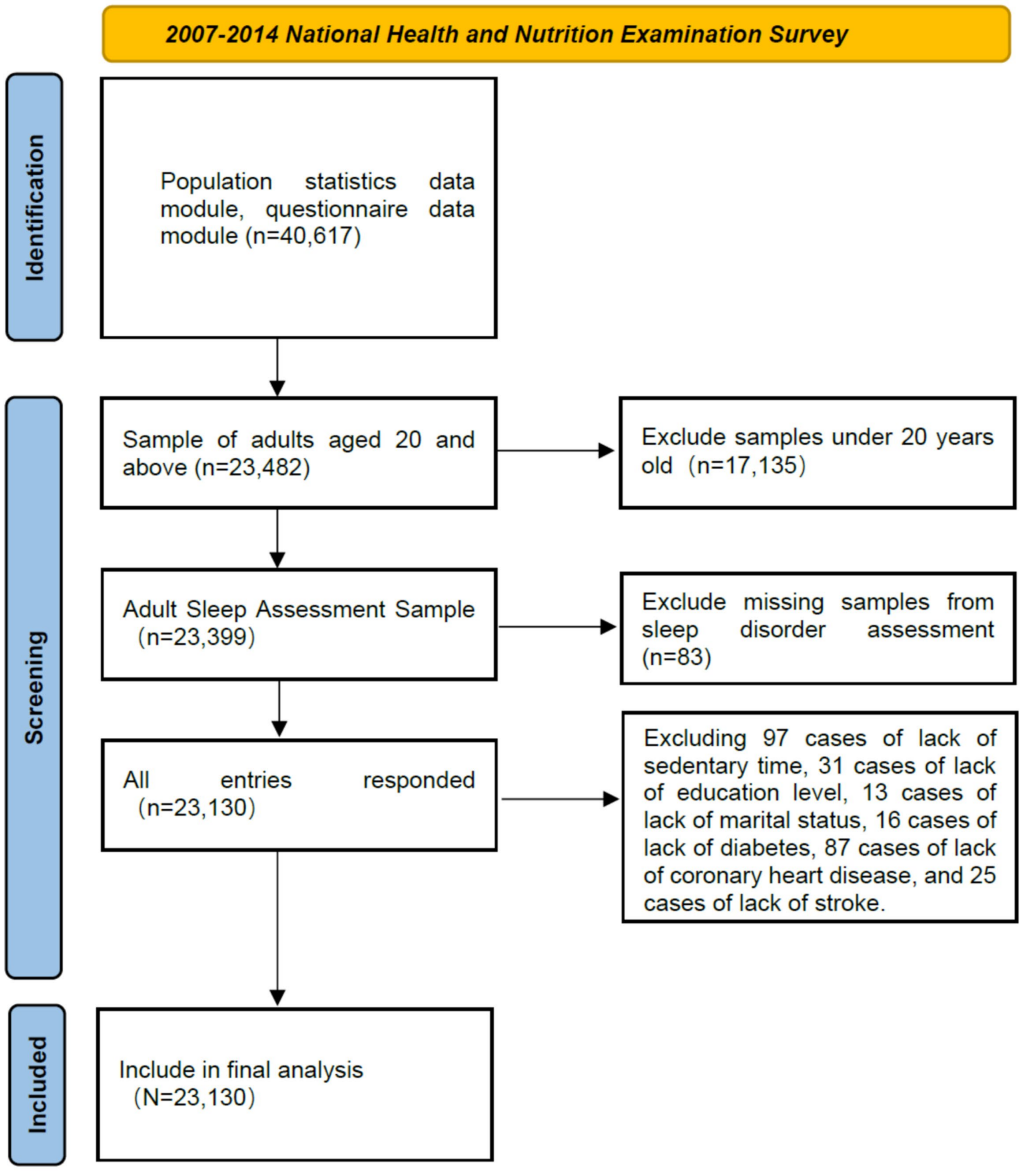
Process diagram for sample inclusion in the study.

Data were obtained from the 2007–2014 National Health and Nutrition Examination Survey (NHANES), a biennial cross-sectional survey collecting demographic, socioeconomic, nutritional, and health information through interviews, physical examinations, and laboratory tests. NHANES is renowned for its high-quality data. We analyzed variables from the 2007–2014 surveys, including participants aged ≥20 years with complete data. The final sample comprised 23,130 eligible individuals (Figure 1).

### 2.2 Study variables and definitions

- Sleep disorders: Defined as physician-diagnosed sleep disorders, recorded as “yes” or “no” in NHANES.
- Sedentary duration: Categorized into quartiles: Q1 (<180 min), Q2 (180–<300 min), Q3 (300–<480 min), Q4 (≥480 min).
- Sleep duration: Grouped as <6 h, 6–<8 h, and ≥8 h.
- Covariates: Demographic (age, sex, education level, marital status) and health-related (coronary heart disease, stroke, diabetes).

### 2.3 Statistical analysis

Perform statistical analysis using R version 4.3.0. Classify the data as n (%) and use chi square test for inter group comparison. Use binary logistic regression to analyze the relationship between sedentary behavior, sleep duration, and sleep disorders. Model 1 did not adjust for any variables; Model 2 has been adjusted based on significant differences in social and demographic characteristics; Model 3 has been adjusted based on demographic characteristics and chronic diseases. Perform subgroup analysis by age and gender to explore differences between subgroups. Use restricted cubic spline (RCS) to analyze the dose–response relationship between sedentary behavior, sleep duration, and sleep disorders. The results are expressed as the odds ratio (OR) of the 95% confidence interval (CI), and when *p* < 0.05, the difference is considered statistically significant.

## 3 Results

### 3.1 Baseline characteristics

The study included 23,130 adults (mean age: 49.49 ± 17.80 years; 48.57% male, 51.43% female). Significant differences (*p* < 0.05) were observed between groups with and without sleep disorders in sedentary duration, sleep duration, age, sex, education level, marital status, and chronic conditions (Table 1).

**Table 1:** Comparison of general characteristics of adults with and without sleep disorders.

Variables	Total (*n* = 23,130)	Non sleep disorder group (*n* = 21,206)	Sleep disorders group (*n* = 1,924)	Statistic	*p*
Sedentary activity, *n* (%)				*χ*^2^ = 76.584	**<0.001**
Q1	3,963 (17.13)	3,720 (17.54)	243 (12.63)		
Q2	5,615 (24.28)	5,240 (24.71)	375 (19.49)		
Q3	5,751 (24.86)	5,213 (24.58)	538 (27.96)		
Q4	7,801 (33.73)	7,033 (33.17)	768 (39.92)		
Sleep (h), *n* (%)				*χ*^2^ = 319.864	**<0.001**
<6 h	3,601 (15.57)	3,032 (14.30)	569 (29.57)		
6–<8 h	11,586 (50.09)	10,733 (50.61)	853 (44.33)		
≥8 h	7,943 (34.34)	7,441 (35.09)	502 (26.09)		
Age, *n* (%)				*χ*^2^ = 155.841	**<0.001**
<45	9,853 (42.60)	9,288 (43.80)	565 (29.37)		
45–<60	5,607 (24.24)	4,996 (23.56)	611 (31.76)		
≥60	7,670 (33.16)	6,922 (32.64)	748 (38.88)		
Gender, *n* (%)				*χ*^2^ = 8.316	**0.004**
Male	11,234 (48.57)	10,239 (48.28)	995 (51.72)		
Female	11,896 (51.43)	10,967 (51.72)	929 (48.28)		
Education level, *n* (%)				*χ*^2^ = 16.758	**0.002**
Less than 9th grade	2,530 (10.94)	2,350 (11.08)	180 (9.36)		
9–11th grade (Includes 12th grade with no diploma)	3,557 (15.38)	3,271 (15.42)	286 (14.86)		
High school graduate/GED or equivalent	5,281 (22.83)	4,840 (22.82)	441 (22.92)		
Some college or AA degree	6,606 (28.56)	5,988 (28.24)	618 (32.12)		
College graduate or above	5,156 (22.29)	4,757 (22.43)	399 (20.74)		
Marital status, *n* (%)				*χ*^2^ = 78.424	**<0.001**
Married	11,821 (51.11)	10,846 (51.15)	975 (50.68)		
Widowed	1,960 (8.47)	1,792 (8.45)	168 (8.73)		
Divorced	2,520 (10.89)	2,220 (10.47)	300 (15.59)		
Separated	778 (3.36)	692 (3.26)	86 (4.47)		
Never married	4,330 (18.72)	4,063 (19.16)	267 (13.88)		
Living with partner	1,721 (7.44)	1,593 (7.51)	128 (6.65)		
Coronary heart disease, *n* (%)				*χ*^2^ = 132.683	**<0.001**
No	22,203 (95.99)	20,451 (96.44)	1,752 (91.06)		
Yes	927 (4.01)	755 (3.56)	172 (8.94)		
Stroke, *n* (%)				*χ*^2^ = 85.240	**<0.001**
No	22,245 (96.17)	20,469 (96.52)	1,776 (92.31)		
Yes	885 (3.83)	737 (3.48)	148 (7.69)		
Diabetes, *n* (%)				*χ*^2^ = 356.759	**<0.001**
No	19,791 (85.56)	18,423 (86.88)	13,68 (71.10)		
Borderline	500 (2.16)	410 (1.93)	90 (4.68)		
Yes	2,839 (12.27)	2,373 (11.19)	466 (24.22)		

Bold values indicates *p* < 0.05.

### 3.2 Multivariate logistic regression

The first logistic regression model only included Sedentary activity or Sleep duration. The results showed that the third quartile of Sedentary activity duration [OR = 1.580 (95% CI: 1.350–1.849), *p* < 0.05] and the fourth quartile [OR = 1.672 (95% CI: 1.439–1.942), *p* < 0.05] had an increased risk of developing sleep disorders in adults relative to the first quartile. Adults with sleep duration of 6–<8 h [OR = 0.423 (95% CI: 0.378–0.474), *p* < 0.05] and ≥8 h [OR = 0.359 (95% CI: 0.317–0.408), *p* < 0.05] have a lower risk of developing sleep disorders compared to those with sleep duration of <6 h.

The second logistic regression model adjusted for variables with significant differences in demographic characteristics (age, gender, education level, marital status), and the results showed that the third and fourth percentiles of sedentary activity duration [OR = 1.528 (95% CI: 1.302–1.794), *p* < 0.05], and the fourth quartile [OR = 1.616 (95% CI: 1.385–1.885), *p* < 0.05] had an increased risk of sleep disorders compared to the first quartile of adults. Adults with sleep duration of 6–<8 h [OR = 0.432 (95% CI: 0.385–0.485), *p* < 0.05] and ≥8 h [OR = 0.368 (95% CI: 0.324–0.419), *p* < 0.05] have a lower risk of developing sleep disorders compared to those with sleep duration of <6 h.

The third logistic regression model adjusted for all significant differences in demographic characteristics (age, gender, education level, marital status) and chronic diseases (Coronary heart disease, stroke, diabetes). The results showed that the third and fourth percentiles of sedentary activity duration [OR = 1.441 (95% CI: 1.226–1.693), *p* < 0.05] and the fourth quartile [OR = 1.480 (95% CI: 1.267–1.729), *p* < 0.05] had an increased risk of developing sleep disorders in adults relative to the first quartile. Sleep duration 6–<8 h [OR = 0.444 (95%CI: 0.395–0.499), *p* < 0.05], ≥8 h [OR = 0.370 (95%CI: 0.325–0.422), *p* < 0.05] the risk of sleep disorders decreases in adults with a relative duration of less than 6 h.

In binary logistic regression analysis, confounding variables may be associated with sedentary behavior, sleep duration, and the onset of sleep disorders, which may lead to biased results. Therefore, this study will construct three logistic regression models to evaluate the robustness of the results by sequentially excluding these confounding factors. Sensitivity analysis confirms that the relationship between sedentary behavior, sleep duration, and sleep disorders remains close (Table 2).

**Table 2:** Multivariate logistic regression analysis of sedentary behavior, sleep duration, and sleep disorders.

Variable	Subgroup	Model 1	Model 2	Model 3
Sedentary activity				
	Q1	1.000 (Reference)	1.000 (Reference)	1.000 (Reference)
	Q2	1.096 (0.927–1.294)	1.051 (0.888–1.245)	1.029 (0.869–1.219)
	Q3	1.580* (1.350–1.849)	1.528* (1.302–1.794)	1.441* (1.226–1.693)
	Q4	1.672* (1.439–1.942)	1.616* (1.385–1.885)	1.480* (1.267–1.729)
Sleep (h)				
	<6	1.000 (Reference)	1.000 (Reference)	1.000 (Reference)
	6–<8	0.423* (0.378–0.474)	0.432* (0.385–0.485)	0.444* (0.395–0.499)
	≥8	0.359* (0.317–0.408)	0.368* (0.324–0.419)	0.370* (0.325–0.422)

*p < 0.05; Model 1 did not adjust for any variables; Model 2 incorporates analysis variables with significant differences in demographic characteristics (age, gender, education level, marital status) for adjustment; Model 3 adjusted for significant differences in demographic characteristics (age, gender, education level, marital status) and chronic diseases (Coronary heart disease, Stroke, Diabetes).

Further subgroup analysis of the association between sedentary behavior, sleep duration, and sleep disorders (Table 3). The results of the third logistic regression model showed that compared with the first quartile of Sedentary activity duration in age subgroups <45 years old, 45–<60 years old, and ≥60 years old, fourth quartiles increased the risk of sleep disorders (all OR > 1, all *p* < 0.05) The risk of sleep disorders is higher in the fourth quartile of sedentary behavior among the 45 year old population.

**Table 3:** Logistic regression table of the relationship between sedentary behavior, sleep duration, and sleep disorders in subgroups.

Variable	Sedentary activity	Model 1	Model 2	Model 3	Sleep (h)	Model 1	Model 2	Model 3
Age								
<45								
	Q1	1.000 (Reference)	1.000 (Reference)	1.000 (Reference)	<6 h	1.000 (Reference)	1.000 (Reference)	1.000 (Reference)
	Q2	1.070 (0.797–1.436)	1.053 (0.783–1.416)	1.042 (0.773–1.404)	6–<8 h	0.337* (0.276–0.411)	0.348* (0.284–0.426)	0.361* (0.294–0.443)
	Q3	1.525* (1.158–2.009)	1.519* (1.147–2.011)	1.496* (1.127–1.985)	≥8 h	0.274* (0.217–0.346)	0.292* (0.230–0.369)	0.309* (0.243–0.391)
	Q4	1.590* (1.224–2.065)	1.610* (1.230–2.108)	1.570* (1.197–2.060)				
45–<60								
	Q1	1.000 (Reference)	1.000 (Reference)	1.000 (Reference)	<6 h	1.000 (Reference)	1.000 (Reference)	1.000 (Reference)
	Q2	1.001 (0.748–1.340)	0.986 (0.734–1.324)	0.936 (0.695–1.261)	6–<8 h	0.420* (0.344–0.513)	0.439* (0.358–0.537)	0.460* (0.375–0.565)
	Q3	1.532* (1.162–2.018)	1.487* (1.122–1.970)	1.322 (0.994–1.758)	≥8 h	0.408* (0.324–0.514)	0.429* (0.340–0.542)	0.442* (0.349–0.560)
	Q4	1.605* (1.240–2.077)	1.588* (1.214–2.078)	1.411* (1.075–1.852)				
≥60								
	Q1	1.000 (Reference)	1.000 (Reference)	1.000 (Reference)	<6 h	1.000 (Reference)	1.000 (Reference)	1.000 (Reference)
	Q2	1.135 (0.852–1.511)	1.100 (0.824–1.468)	1.081 (0.808–1.445)	6–<8 h	0.554* (0.456–0.673)	0.537* (0.440–0.654)	0.544* (0.446–0.665)
	Q3	1.606* (1.224–2.107)	1.577* (1.197–2.077)	1.496* (1.134–1.974)	≥8 h	0.419* (0.340–0.516)	0.411* (0.333–0.507)	0.403* (0.326–0.499)
	Q4	1.718* (1.321–2.233)	1.668* (1.277–2.178)	1.513* (1.156–1.980)				
Gender								
Male								
	Q1	1.000 (Reference)	1.000 (Reference)	1.000 (Reference)	<6 h	1.000 (Reference)	1.000 (Reference)	1.000 (Reference)
	Q2	1.301* (1.016–1.665)	1.183 (0.922–1.518)	1.178 (0.916–1.514)	6–<8 h	0.461* (0.394–0.540)	0.453* (0.386–0.533)	0.460* (0.390–0.542)
	Q3	1.938* (1.531–2.454)	1.765* (1.389–2.242)	1.686* (1.325–2.147)	≥8 h	0.358* (0.298–0.430)	0.349* (0.290–0.420)	0.346* (0.286–0.418)
	Q4	2.151* (1.718–2.694)	1.938* (1.538–2.441)	1.780* (1.410–2.247)				
Female								
	Q1	1.000 (Reference)	1.000 (Reference)	1.000 (Reference)	<6 h	1.000 (Reference)	1.000 (Reference)	1.000 (Reference)
	Q2	0.942 (0.749–1.185)	0.963 (0.764–1.215)	0.933 (0.739–1.178)	6–<8 h	0.384* (0.326–0.453)	0.410* (0.348–0.484)	0.425* (0.359–0.502)
	Q3	1.321* (1.068–1.635)	1.370* (1.101–1.704)	1.289* (1.035–1.605)	≥8 h	0.361* (0.303–0.432)	0.392* (0.327–0.469)	0.397* (0.331–0.476)
	Q4	1.327* (1.084–1.625)	1.366* (1.108–1.685)	1.265* (1.024–1.563)				

*p < 0.05; Model 1 did not adjust for any variables; Model 2 incorporates analysis variables with significant differences in demographic characteristics (age, gender, education level, marital status) for adjustment; Model 3 adjusted for significant differences in demographic characteristics (age, gender, education level, marital status) and chronic diseases (Coronary heart disease, Stroke, Diabetes).

Adults aged <45, 45–<60, and ≥60 years who sleep for 6–<8 h [all OR<1, all *p* < 0.05] and ≥8 h [all OR < 1, all *p* < 0.05] have a lower risk of developing sleep disorders compared to adults aged <6 h The risk of sleep disorders is higher in the fourth quartile of sedentary behavior among the 45 year old population 6–<8 h, The risk of sleep disorders decreases even more after 8 h.

In the gender subgroups of males and females, compared with those with Sedentary activity duration in the first quartile, both the third and fourth quartiles increased the risk of sleep disorders (all OR > 1, all *p* < 0.05). Men with sedentary behavior have a higher risk of sleep disorders in the third and fourth quartiles.

Among males and females, adults with sleep duration of 6–<8 h [OR < 1, *p* < 0.05] and ≥8 h [OR < 1, *p* < 0.05] have a lower risk of developing sleep disorders compared to adults with sleep duration less than 6 h. Sensitivity analysis within subgroups confirmed that the relationship between sedentary behavior, sleep duration, and sleep disorders remains close.

### 3.3 Restrictive cubic spline curves of sedentary behavior, sleep duration, and sleep disorders

**Figure 2:**
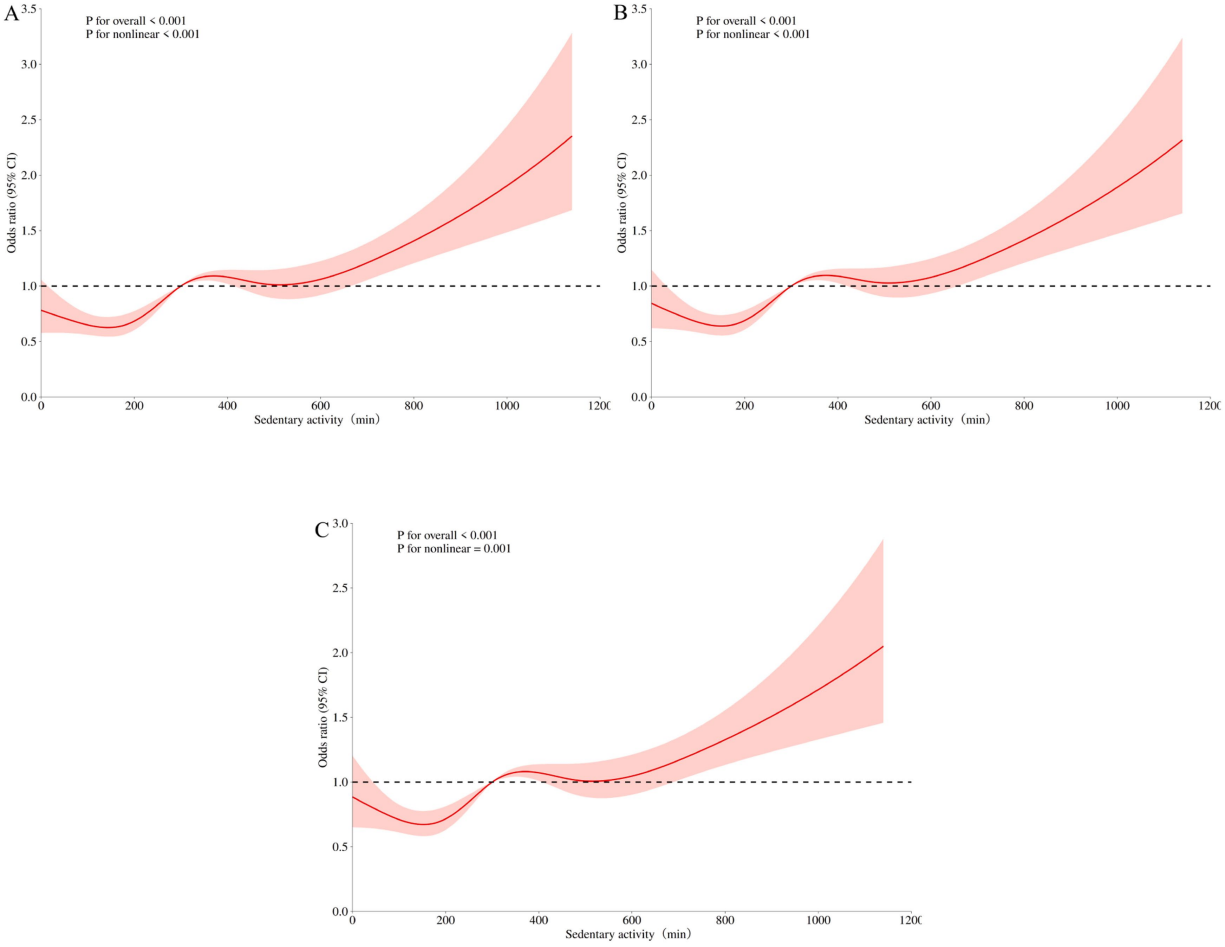
Restricted cubic spline curve of sedentary time and sleep disorders. **(A)** RCS 1 did not adjust for any variables; **(B)** RCS 2 incorporates analysis variables with significant differences in demographic characteristics (age, gender, education level, marital status) for adjustment; **(C)** RCS 3 adjusted for significant differences in demographic characteristics (age, gender, education level, marital status) and chronic diseases (Coronary heart disease, Stroke, Diabetes).

The dose–response relationship between sedentary time and sleep disorders showed a non-linear curve (*P*_overall_ < 0.001, *P*_nonlinear_ = 0.001) (Figure 2C). As sedentary time increased, the risk of sleep disorders showed an increasing trend, and at 300 min, the risk of sleep disorders significantly increased. In RCS analysis, confounding variables may affect the dose–response relationship between sedentary time and the onset of sleep disorders. The study will evaluate the robustness of the results by sequentially excluding these confounding factors. Sensitivity analysis confirms that the relationship between sedentary time and sleep disorders remains close (Figures 2A, B).

**Figure 3:**
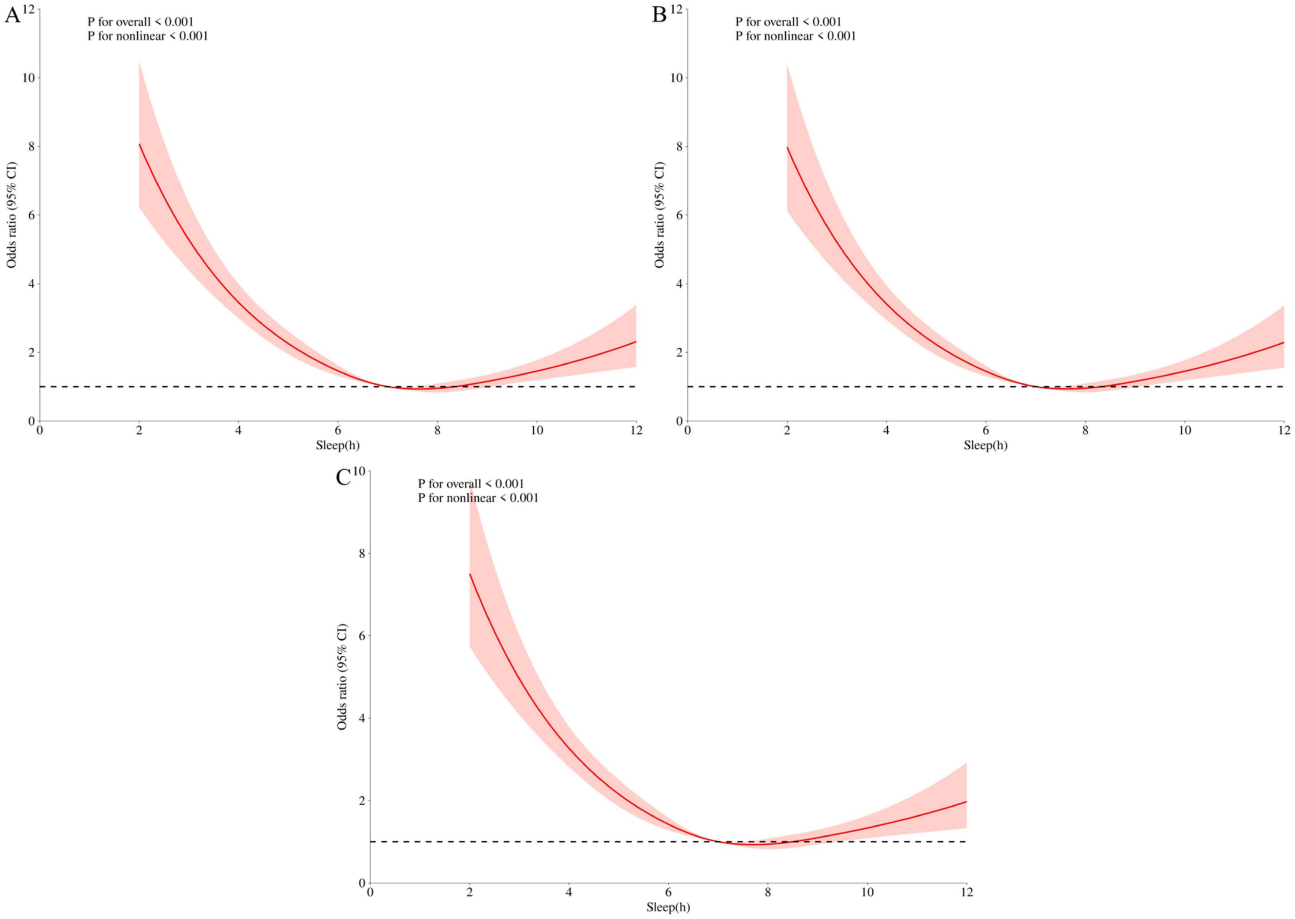
Sleep duration and sleep disorder restrictive cubic spline curve. **(A)** RCS 1 did not adjust for any variables; **(B)** RCS 2 incorporates analysis variables with significant differences in demographic characteristics (age, gender, education level, marital status) for adjustment; **(C)** RCS 3 adjusted for significant differences in demographic characteristics (age, gender, education level, marital status) and chronic diseases (Coronary heart disease, Stroke, Diabetes).

The dose–response relationship between sleep duration and sleep disorders showed a non-linear curve (*P*_overall_ < 0.001, *P*_nonlinear_ < 0.001) (Figure 3C). As sleep duration increased, the risk of developing sleep disorders gradually decreased, and at 7 h, the risk of developing sleep disorders was significantly reduced. It reaches its lowest point around 8 h and shows an upward trend after 8 h. In RCS analysis, confounding variables may affect the dose–response relationship between sleep duration and the onset of sleep disorders. The study will evaluate the robustness of the results by sequentially excluding these confounding factors. Sensitivity analysis confirms that the relationship between sleep duration and sleep disorders remains close (Figures 3A, B).

## 4 Discussion

Good sleep quality plays an important role in physical health. Effective sleep duration not only ensures the normal needs of the body, but also accelerates the recovery of physical and mental strength (32–36). Sleep disorders can lead to disrupted sleep patterns and disrupted regular daily activities, resulting in decreased mental energy and vitality, increased fatigue and sleep disorders (37, 38). They may also impair cognitive function (35, 36) and affect physical and mental health (39–41).

The NHANES questionnaire survey is a commonly used research method that collects a large amount of data to analyze people’s work habits, lifestyle, and sleep conditions, providing rich data support for research (42). This study conducted empirical analysis based on NHANES data, and the results showed a significant correlation between sedentary time, sleep duration, and sleep disorders, which is consistent with the findings of Heiland et al. (43–47). There is a non-linear dose–response relationship between sedentary time and sleep disorders. The risk of sleep disorders in adults increases with time after sedentary time reaches 300 min, which is consistent with the findings of Koohsari et al. (48–51). The reasons why prolonged sitting increases the risk of sleep disorders involve multiple physiological, psychological, and behavioral mechanisms. From a physiological mechanism analysis: Prolonged sitting leads to a decrease in bodily functions, especially affecting metabolism and blood circulation, all of which directly affect sleep quality. Sitting still for a long time can also reduce cerebral blood flow and affect areas related to sleep wake regulation, such as the hypothalamus and brainstem. Metabolic slowdown may also induce insulin resistance and obesity, indirectly exacerbating the risk of sleep apnea. Secondly, prolonged sitting is accompanied by stress reactions and disrupted cortisol secretion, leading to shallow sleep and early awakening. Prolonged sitting can also promote the release of pro-inflammatory cytokines such as IL-6 and TNF-α, and chronic low-grade inflammation is associated with fragmented sleep and reduced deep sleep. From the perspective of biological clock and neural regulation mechanisms, prolonged mental stress associated with prolonged sitting can activate the sympathetic nervous system, inhibit the relaxation state dominated by the parasympathetic nervous system at night, and manifest as difficulty falling asleep and sleep maintenance disorders (52)‌. From the perspective of psychological and behavioral mechanisms, prolonged sitting is often associated with high-intensity work and long screen time. Continuous psychological stress can lead to excessive sympathetic nervous system stimulation, causing insomnia or sleep maintenance disorders (53, 54). Sedentary sitting leads to insufficient exercise, which directly affects sleep. Exercise improves sleep by increasing the accumulation of adenosine and regulating serotonin levels. Sedentary individuals lack pathways for exercise regulation. Sedentary individuals are more likely to continue sitting at night (such as using their phones or watching TV), delaying bedtime and disrupting their sleep rhythm. From the perspective of physical discomfort: Fixed posture leads to continuous compression of the cervical and lumbar vertebrae, muscle stiffness or soreness directly interfering with falling asleep. Sedentary sitting may also cause shoulder and neck strain, increasing the frequency of turning over or adjusting posture during sleep. Sedentary sitting can also lead to reduced venous reflux in the lower limbs, causing restless leg syndrome or nighttime leg cramps, disrupting sleep continuity. From the perspective of the interaction between society and the environment, prolonged sitting reduces exposure to natural light, weakens the adaptability of the biological clock to external light, and leads to abnormal secretion of melatonin and disrupted sleep wake cycles (55, 56)‌. Lack of exercise and social interaction may increase the risk of depression, which is a common comorbidity of sleep disorders. Sleep disorders lead to daytime sleepiness and lack of energy, further reducing activity willingness and exacerbating the interaction between sedentary time and sleep problems (57). In addition, prolonged sitting is often associated with unhealthy eating habits, such as high calorie and high-fat food intake, which may also affect sleep. From a psychological perspective, prolonged periods of static living reduce physical activity, which may affect an individual’s mental health status, such as increasing the risk of anxiety and depression, which are direct causes of sleep disorders (58–60).

As age increases, the duration and quality of nighttime sleep decrease, which is consistent with previous research results (61–63). Further age subgroup analysis revealed that the fourth quartile of sedentary behavior in the middle-aged (<45) population has a higher risk of sleep disorders. In the middle-aged stage, excessive anxiety and tension may occur due to work competition, family responsibilities, etc., leading to difficulty falling asleep or maintaining sleep (64). Women approaching menopause (around 45 years old) may experience fluctuations in sex hormone levels, causing autonomic nervous system disorders and sleep problems (65). Gender subgroup analysis found that there are differences in the impact of sedentary time on sleep disorders between males and females. Males are more likely to experience sleep disorders, which may be related to physiological factors such as hormone levels and circadian rhythms, as well as psychological factors such as social roles and stress. The gradual decrease of testosterone in middle-aged men may cause metabolic disorders and autonomic nervous system disorders, indirectly leading to difficulty falling asleep or fragmented sleep. Men generally bear the burden of family economic support and social competition pressure, and long-term anxiety and tension can easily lead to chronic insomnia. Men are less likely to actively release negative emotions, and the accumulation of psychological stress may transform into somatic symptoms (such as insomnia). Excessive drinking, smoking, caffeine dependence, and other behaviors in men directly affect the excitability of the nervous system and disrupt sleep rhythms.

This study has certain limitations: firstly, it used cross-sectional surveys from 2007 to 2014, which cannot confirm the causal relationship between sedentary time, sleep duration, and sleep disorders; Secondly, sleep disorders are not only influenced by lifestyle habits, but also by factors such as living environment, social environment, family relationships, and economic level. Looking forward to future research continuing to focus on the relationship between sedentary time and sleep disorders, particularly investigating sleep fragmentation, sleep interruption time, sleep rhythms, and exploring more effective interventions to improve this issue.

## 5 Conclusion

In summary, there is a clear non-linear relationship between sedentary time, sleep duration, and sleep disorders. Therefore, it is recommended to limit sedentary time to 300 min per day, and to get up and move for 5–10 min every hour to engage in simple stretching or walking exercises to promote blood circulation and reduce health risks caused by prolonged sitting. It is recommended to engage in at least 30 min of moderate intensity or 15 min of high-intensity physical activity per day to improve sleep quality, and a recommended sleep duration of 7–8 h to reduce the risk of sleep disorders.

## Data Availability

The raw data supporting the conclusions of this article will be made available by the authors without undue reservation.
